# A brief guide to writing a medical physics leadership case

**DOI:** 10.1002/acm2.13186

**Published:** 2021-03-19

**Authors:** Dongxu Wang, Leonard Kim, Mary Gronberg, Cassandra Stambaugh, AAPM Medical Physics Leadership Academy (MPLA) Cases Subcommittee

**Affiliations:** ^1^ Memorial Sloan Kettering Cancer Center New York NY USA; ^2^ MD Anderson Cancer Center at Cooper Camden NJ USA; ^3^ University of Texas MD Anderson Cancer Center Houston TX USA; ^4^ Tufts Medical Center Boston MA USA

**Keywords:** case study, leadership, MPLA

This guide provides a framework and general steps for writing a case study for the Medical Physics Leadership Academy (MPLA).^1^, ^2^ This guide may be used as part of the Request for Proposal (RFP) for case studies in AAPM leadership‐themed sessions.

## 1 **Step 1.** Develop a scenario and learning objectives

The first step in writing a case study is identifying learning objectives and developing a scenario. You may already have a scenario in mind before having considered learning objectives. Remember that a case study is not just telling a personal story related to leadership. It is an educational tool that should be specifically aligned to the MPLA's curriculum (available at https://w3.aapm.org/leadership/curriculum.php). Identifying your learning objectives up‐front will help you focus your scenario and develop discussion points (see Step 3), as will decide the category of your case. The MPLA uses categories defined in William Ellet's *The Case Study Handbook* ^3^:

1. Decision Scenario Case: The case presents a “need to make a critical decision and potentially persuade” others to accept it.
2. Evaluation Scenario Case: The case presents a “need to perform an in‐depth evaluation that lays out the pros and cons”
3. Problem‐Diagnosis Scenario Case: The case presents a “need to perform a comprehensive problem diagnosis that identifies the root causes of a problem”

Consideration of the case category and learning objectives should be maintained while developing the case synopsis, case study and facilitators guide.

## 2 Step 2. Develop a case synopsis

A case synopsis is a high‐level summary of the case study. An example of a case synopsis is given in Exhibit 1 in *Supplemental Materials*. The following components should be included: *setting*, including information about technology or organizational structure when needed to understand the case; *plot*, an explanation of the situation; *discussion points*; and *characters*, including job titles, personality, beliefs, and concerns. Remember that your case study is a work of fiction. Even when inspired by a real‐life event, **names of characters, locations, and institutions should all be invented**. (You may use actual product and vendor names if doing so will not influence the discussion.)

Developing a synopsis will help you evaluate the appropriateness of your setting, plot, and characters before writing the case study. The example synopsis in Exhibit 1 allowed the authors to identify a lack of diversity in the characters and rework them in order to reach a broader audience. It also allowed them to eliminate characters that took focus away from the learning objectives.

Outlining discussion points in your synopsis will help determine what details to include in your final version. In this example, the author wanted readers to identify IT and billing as potential factors in decision‐making, and identifying these factors as discussion points enabled the authors to allude to them in the final version of *MPLA Case 1: Implementing Cone‐Beam CT in a Community Hospital*.^2^ without explicitly stating them. An effective case study should not have clear, black‐and‐white solutions, but should inspire discussion and the consideration of multiple competing factors. A good synopsis will help you with writing the case, as well as assist reviewers of your submission.

## 3 Step 3. Develop the case and facilitator's guide

An MPLA case should be **no more than 2,500 words**, roughly four to five single‐spaced pages using 12‐point font size. A reader should be able to finish the case study within 15‐20 minutes to accommodate sufficient discussion time if such a case is used in a typical hour‐long meeting or discussion session. For those without much experience in creative writing, seek help from expert writers and literary enthusiasts. “*MPLA Case 1: Implementing Cone Beam CT in a Community Hospital*”^2^ was written with the assistance of an English and creative writing major who worked from the case synopsis and professional case studies (non‐medical physics) as models. Seek help from experts when writing about non‐medical physics areas. For example, a case study about purchasing new equipment may require assistance in obtaining and presenting realistic financial and budgetary data.

The facilitator's guide is a teaching note that guides instructors on how to use your case study to facilitate discussion. It should be written in parallel with the case study and stay consistent to the scenario case study defined in Step 1. See *Sample Facilitator's Guide for* “*MPLA Case 1. Implementing Cone Beam CT in a Community Hospital*”^2^ for a model of how to write a facilitator's guide. The MPLA Cases Subcommittee will publish a guide on writing a facilitator guide.

## 4 Step 4. Review and revise

Your case study needs to be relatable and discussion‐worthy. Revisit step 1 to ensure your case study retains its real‐life essence while clearly advancing your learning objectives. Seek feedback on whether desired discussion points arise naturally from your case study presentation. The MPLA Cases Subcommittee offers some support for reviewing and revising new case studies. Please reach out to the Subcommittee if you have any questions. Our contact information can be found from AAPM's website. Thank you for contributing to the MPLA's mission and have fun writing!

## Supporting information

Supplementary Material
