# Supplementary material for: A brief guide to writing a medical physics leadership case
Source: J Appl Clin Med Phys. 2021 Mar 19;22(3):285–6. doi: 10.1002/acm2.13186 (PMC7984477; doi:10.1002/acm2.13186)
Supplement: Supplementary file 1 — Supplementary Material [file ACM2-22-285-s001.docx]

**Exhibit 1: MPLA Case 1 Original Synopsis and Suggested Discussion Points**

“Implementing cone beam CT in a community hospital” by Dongxu Wang.

***Setting****: This is a community hospital in a rural Midwest town with population of 5,000 ~ 10,000 people. The radiation therapy treatment in the cancer clinic is provided by a Varian 21EX, a once-workhorse, now-outdated linear accelerator. Nevertheless, it is equipped with on-board imager and capable of delivering the standard care of three-dimensional image guided radiation therapy (IGRT) with cone-beam computed tomography (CBCT), except that it is not being used.*

***Plot****: Dr. Garner joined the practice as the solo medical physicist, overseeing technology in the clinic. He was surprised to see that CBCT is not used; he thought it should be as universal as XXX (name a universal but funny thing?). He began to talk to everyone in the clinic, trying to figure out why, and most importantly, how to implement CBCT. He believes he is serving the best interest of the patients here.*

*Upon inquiry, Garner learned that the physicians’ office computers were not powerful enough to review CBCT and crashed whenever trying load CBCT. He further learned that the clinical supervisor was wary of any additional capital investment on computers, while the hospital IT department had its own policy regarding the specifications of office workstations. Dr. Garner really wanted to upgrade or buy new computers to utilize CBCT for the best of patient care. One physician was fully supportive of this initiative; the other showed disinterest, but not discouragement. Utilizing CBCT, meanwhile, means workflow changes to medical dosimetrist and radiation therapists that require some training and education. Garner believed that would be easily manageable, but that everyone was convinced.*

*What can Dr. Garner do? Please have a discussion and lay out some key points.*

***Discussion Point*** *1 – On financial side, IGRT billing charges were not fully captured if CBCT were not utilized. You could research a little bit of the lost revenue, and come up with a financial pro forma to show to the department administrator. It should be reasonable to expect that the captured IGRT charges with CBCT can easily cover the cost of purchasing the new computers. But this requires the hospital billing staff to learn and apply the new charges.*

***Discussion Point*** ***2*** *– On technical side, it is worth discussing with the hospital IT the difference between office computer and medical equipment. You should request a vendor statement regarding the recommended computer configuration for the best workstation performance. You could request the hospital IT to consider the CBCT workstations to be medical equipment and be exempted from their general policy regarding office computers. However, this may not be agreed upon by hospital IT.*

***Discussion Point 3*** *– Different members of the clinic have different personalities and incentives. It is critical to learn how to sell the new idea to each colleague and lead the overall change.*

***Potential Characters:***

*Jon Garner, PhD, 35 yo, Medical Physicist. Newly recruited as the solo medical physicist; former colleague of Dr. Mitchell in previous institution. Interested in more leadership experience in a community hospital setting instead of working in a large group. Also appreciates small town living. Wants to make a difference!*

*Aaron Mitchell, MD, 31 yo, Junior Radiation Oncologist. Newly minted from a major medical school’s residency program in a big city. After 13 years, he is happy to return to the region near his hometown and be close to his family, but he is not satisfied with the status of the medical practice he is in. And, he is not quite sure how to change the status and his relationship to the senior physician in this practice, who was his mentor and clearly hired him in the first place.*

*Paul Bell, MD, 58 yo. Senior Radiation Oncologist. Likes the old way; not tech-savvy; waiting to retire. did not believe there are new clinical evidence for IGRT anyway.*

*Mary Robinson, CMD, 40 yo, Medical Dosimetrist. Having been here for 18 years since college. Although educated in the old way, she is eager and quick to learn new things. On the other hand, she clearly sees her position in the managerial hierarchy and does not want to be the one initiating changes. Obedient and extremely well-tempered.*

*Jane Mills, RTT, 50 yo, Senior Radiation Therapist, Clinical Supervisor. Dislike changes, and suspicious of all the new things people are bringing up (especially by those from a very different background – “they don’t know our Midwest small town way”!). Very afraid of changes, especially in her daily procedural workflows.*

*McKenzie Wilson, 23 yo, RTT, Junior Radiation Therapist. Lives one hour away in a much bigger city and trained there. Happy to have a not-so-demanding job here, but often wonders if she is too content with status quo.*

*Heather Highland, BS, 30 yo, off-site biller. Capable of what she is doing, but may not be best informed in radiation oncology-specific charges.*

*Tom Wayne, 55 yo, Vendor representative. Knowledge and supportive, even though is cancer center is only a small account of his in the region he oversees.*
